# Endoscopic ultrasound-guided biliary drainage: a literature review

**DOI:** 10.1590/0100-6991e-20233414-en

**Published:** 2023-03-13

**Authors:** RODRIGO RODA RODRIGUES DA-SILVA, LUCAS GALLO DE ALVARENGA MAFRA, VITOR OTTOBONI BRUNALDI, LETÍCIA FRANÇA DE ALMEIDA, EVERSON LUIZ DE ALMEIDA ARTIFON

**Affiliations:** 1 - Hospital Mater Dei Santo Agostinho, Serviço de Endoscopia Digestiva - Belo Horizonte - MG - Brasil; 2 - Hospital das Clínicas da UFMG, Instituto Alfa de Gastroenterologia do Hospital das Clínicas da UFMG - Belo Horizonte - MG - Brasil; 3 - Hospital das Clínicas de Ribeirão Preto, Departamento de Cirurgia e Anatomia, Centro de Endoscopia - Ribeirão Preto - SP - Brasil; 4 -Hospital das Clínicas da Faculdade de Medicina da USP, Departamento de Gastroenterologia, Unidade de Endoscopia Gastrointestinal - São Paulo - SP - Brasil; 5 - Universidade de São Paulo, Departamento de Cirurgia Geral - São Paulo - SP - Brasil

**Keywords:** Endosonography, Choledochostomy, Biliary Tract Neoplasms, Stents, Cholestasis, Endossonografia, Coledocostomia, Neoplasias do Sistema Biliar, Stents, Colestase

## Abstract

Neoplasms of the biliopancreatic confluence may present with obstruction of the bile tract, leading to jaundice, pruritus and cholangitis. In these cases drainage of the bile tract is imperative. Endoscopic retrograde cholangiopancreatography (ERCP) with placement of a choledochal prosthesis is an effective treatment in about 90% of cases, even in experienced hands. In cases of ERCP failure, therapeutic options traditionally include surgical bypass by hepaticojejunostomy (HJ) or percutaneous transparietohepatic drainage (DPTH). In recent years, endoscopic ultrasound-guided biliary drainage techniques have gained space because they are less invasive, effective and have an acceptable incidence of complications. Endoscopic echo-guided drainage of the bile duct can be performed through the stomach (hepatogastrostomy), duodenum (choledochoduodenostomy) or by the anterograde drainage technique. Some services consider ultrasound-guided drainage of the bile duct the procedure of choice in the event of ERCP failure. The objective of this review is to present the main types of endoscopic ultrasound-guided biliary drainage and compare them with other techniques.

## RESUMO

Neoplasias da confluência biliopancreática podem cursar com obstrução da via biliar, levando a icterícia, prurido e colangite. Nesses casos a drenagem da via biliar é imperativa. A colangiopancreatografia endoscópica retrógrada (CPER) com colocação de prótese coledociana constitui tratamento eficaz em cerca de 90% dos casos mesmo em mãos experientes. Nos casos de insucesso da CPER, tradicionalmente as opções terapêuticas incluem a derivação cirúrgica por hepaticojejunostomia (HJ) ou drenagem percutânea transparietohepática (DPTH). Nos últimos anos as técnicas endoscópicas ecoguiadas de drenagem biliar ganharam espaço por serem menos invasivas, eficazes e apresentarem incidência aceitável de complicações. A drenagem endoscópica ecoguiada da via biliar pode ser realizada pelo estômago (hepatogastrostomia), duodeno (coledocoduodenostomia) ou pela técnica de drenagem anterógrada. Alguns serviços consideram a drenagem ecoguiada da via biliar o procedimento de escolha no caso de insucesso da CPER. O objetivo desta revisão é apresentar os principais tipos de drenagem biliar endoscópica ecoguiada e confrontá-los com outras técnicas.

## INTRODUCTION

Malignant neoplasms of the biliopancreaticoduodenal confluence, such as neoplasms of the head of the pancreas, duodenal papilla, distal cholangiocarcinomas, and metastatic lesions involving this region, constitute a heterogeneous group of diseases that can culminate in biliary tract obstruction. Such pathologies have a similar clinical picture and treatments, as well as a usually poor prognosis, with low rates of curative surgical resection and of survival^1^ ^,^ ^2^.

Signs and symptoms of malignant obstruction of the biliary tract include cholestasis, with jaundice, choluria, and acholia, pruritus, and possible progression to cholangitis. In view of the potential severity of the condition, clearance or draining of the bile duct is imperative. Currently, endoscopic retrograde cholangiopancreatography (ERCP) is well established as the treatment of choice for clearing the biliary tract, promoting an abrupt drop in bilirubin levels in about 90% of cases^3^ ^,^ ^4^. The 10% of the remaining cases represent situations of ERCP failure in clearing the biliary tract, even using advanced cannulation techniques or after new ERCP attempts^4^ ^,^ ^5^. Such adversities can be divided into those that prevent the progression of the duodenoscope to the second duodenal portion and those that prevent selective cannulation of the biliary tract (Table 1).

**Table 1:** Causes of failure of ERCP in the palliative treatment of neoplasms of the biliopancreatic junction.

Inaccessible Major Duodenal Papilla
• Peptic strictures of the esophagus and duodenal
• Neoplastic duodenal infiltration preventing the progression of the duodenoscopy
• Duodenal stent previously placed
• Surgeries with intestinal transit diversion (ex: esophagus gastrectomy, gastrectomy with roux-en-y reconstruction, bariatric by-pass)
Accessible Major Duodenal Papilla
• Technical difficulty of cannulation (intra or peridiverticular papilla)
• Inability to progress the guidewire proximally to the stenosis
• Gross neoplastic infiltration of the major duodenal papilla

In situations of ERCP failure, established therapeutic possibilities include surgical hepaticojejunostomy (HJ) or percutaneous transparietohepatic drainage (PTHD). However, in 2001, Giovanninni et al.6 described an endoscopic treatment for malignant biliary obstruction with an endoscopic ultrasound-guided choledochoduodenostomy (EUS-CDT), using a 10Fr straight plastic biliary prosthesis. Since then, the endoscopic ultrasound-guided biliary drainage (EUS-BD) has evolved and become an alternative for cases in which ERCP fails to clear the biliary tract.

The objective of this review is to present the three main EUS-BD techniques and compare the results of EUS-CDT with other ultrasound-guided techniques and with the traditional HJ and PTHD. Finally, we aim to evaluate the different models of available prostheses, plastic, metal, and the modern lumen apposition prostheses (LAMS), the later having gained notoriety for their ease of handling, but still showing restricted experience due to their high cost.

## METHODS

The present work is a narrative review, carried out with articles from the main electronic databases. Because it is a review of previously published articles, the work was not appraised by the Ethics in Research Committee (CEP) of the involved institution.

For the selection of articles, we carried out a research in the databases PubMed, SciElo, and Cochrane Library. We used combinations of the terms “ERCP”, “failed ERCP”, “‘choledocho-duodenostomy”, “hepaticogastrostomy”, “biliary tract drainage”, “percutaneous biliary drainage”, “biliary distal obstruction”, “EUS-guided”, and “LAMS”. Due to the large number of techniques, some already classically used and others more recent, we considered articles published in English between 2001 and 2021.

The authors evaluated the articles independently, excluding the ones not purporting to humans and those not related to the ultrasound-guided biliary drainage techniques chosen for the study.

With the pre-established search criteria, we included 45 papers in the review, consisting of original articles, narrative reviews, systematic reviews, and meta-analyses. 

## RESULTS AND DISCUSSION

Endoscopic ultrasound-guided biliary drainage (EUS-BD) can be performed using different techniques, which have shown high rates of clinical success. In 2006, five years after the first described EUS-BD, Kahaleh et al.^9^ reported a series of EUS-BD cases using different techniques, with a 91% success rate in biliary decompression and a 17% complication rate. Two published meta-analyses found rates above 90% of technical success and 17% to 23% of adverse events in patients undergoing EUS-BD^11^ ^,^ ^12^.

A systematic review published by Dhindsa et al.^13^ in 2020 covering 23 studies and totaling 1,437 patients aimed at measuring technical and clinical success rates of the different EUS-BD techniques. The results showed 91.5% technical success and 87% clinical success, in addition to a 17.9% incidence of adverse events, the most frequent being biliary fistula (4%), stent migration (3.9%), and infection (3.8%). This systematic review included studies with different EUS-BD techniques (34.4% undergoing EUS-CDT), different types of stents (metallic, plastic, and metallic with luminal apposition), use or not of a nasobiliary drain, and professionals with different levels of experience, resulting in high heterogeneity (76.5%).

### Ultrasound Guided Hepatogastrostomy (US-HG)

US-HG is one of the possible EUS-BD techniques, being a good alternative for lesions of the hepatic hilum or when the guidewire cannot progress distally to the biliary obstruction.

**Figure 1:**
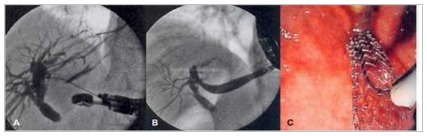
Echoguided hepatogastrostomy. A) cholangiography after ultrasound-guided puncture of the left intrahepatic bile duct; B) release of metallic stent; C) endoscopic view of the stent from the stomach [images courtesy of Artifon ELA].

The technique consists of the following steps: positioning the echoendoscope in the subcardiac region; identifying a dilated bile duct in the left lobe, usually in segment III, and puncturing it with a 19G^14^ needle (a 22G can also be used); performing cholangiography and confirming proper needle positioning with injection of iodinated contrast; passing a hydrophilic guidewire in the biliary tract, preferably keeping it distal to the stenosis (a 0.35” guidewire is recommended for a 19G needle and a 0.25” one for a 22G needle); dilating the path between the stomach and the liver with the chosen accessory (cystotome, progressive rigid dilators - cotton dilator -, stylet, or hydrostatic balloon^14^ - Figure 1), being very careful not to lose the guidewire positioning; inserting a plastic or metal prosthesis in the dilated path to maintain the hepatogastric fistula.

Some specialists suggest the use of a 6Fr or 7Fr nasobiliary drain through the metal prosthesis for a period of 48 hours, with the aim of reducing the chances of early migration of the prosthesis. An alternative with the same purpose consists of introducing a covered metal prosthesis inside an uncovered metal one positioned anteriorly. This strategy aims at better anchorage, with less migration related to the uncovered prosthesis, associated with a tendency towards better drainage and less bile leakage into the cavity related to the coated prosthesis^14^ ^,^ ^15^.

The Giovannini group^7^ published, in 2003, a complex case submitted to US-HG of a biliary tract obstruction with hepatic hilar involvement due to lymphadenopathy in a patient with a history of adenocarcinoma treated with gastrectomy. The patient was initially drained with a metallic prosthesis by PTHD, followed by US-HG with a plastic prosthesis, which was later replaced by a metallic stent. During the five-month follow-up, the patient experienced jaundice relief.

Artifon et al.^10^ published the first report of US-HG using a partially covered metallic prosthesis in 2007.

### Anterograde drainage of the extrahepatic bile duct (rendez-vous)

The eco-guided rendez-vous, presented by Mallery et al.^8^ in 2004, allowed access for drainage in cases with changes in the local anatomy, contributing to the enhancement of EUS-BD.

In the original description, the echoendoscope was used to puncture the Wirsung duct through the gastric window, with subsequent passage of the guidewire to the duodenum. Afterwards, using the duodenoscope, the duodenal guide wire was captured, which, in sequence, was used for cannulation according to the conventional technique.

**Figure 2:**
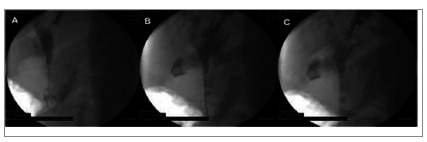
Anterograde drainage in a patient with a history of gastrectomy. A) puncture of the intrahepatic bile duct with cholangiography and subsequent passage of the wire guide below the stenosis; B) placement of the metallic stent until the intestinal loop; C) release of the prosthesis under fluoroscopic view [images courtesy of Artifon ELA].

The adaptation of the technique for puncture of the extrahepatic bile duct by ultrasound endoscopy (access route) and subsequent conventional anterograde drainage is indicated for cases with anatomical deformities or local duodenal infiltration (Figure 2).

The technique used in anterograde drainage is similar to US-HG. However, after dilating the path, the metal stent is released, crossing the stenosis. In distal neoplastic obstructions, the stent may have one end positioned in the duodenum or intestinal loop (in cases with a history of surgical approaches, such as gastrectomy or by-pass).

### Endoscopic ultrasound guided choledochoduodenostomy (EUS-CDT)

**Figure 3:**
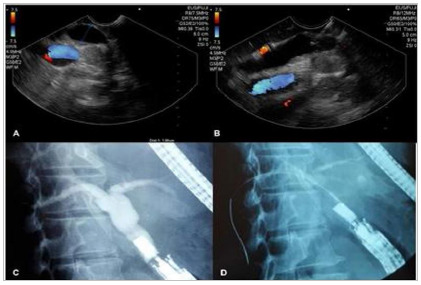
EUS-CDT. A) endosonographic identification of the common bile duct from the duodenal bulb; B) bile duct puncture with a 19 G needle; C) cholangiography confirming access to the biliary tract and showing dilatation of the intra and extrahepatic bile ducts; D) passage of the wire guide until intrahepatic bile duct.

EUS-CDT consists of a EUS-BD technique in which the extrahepatic bile duct is punctured through the duodenal bulb (Figure 3), thus indicated in extrahepatic obstructions with biliary tract dilation and without neoplastic infiltration in the topography of the puncture. Among the advantages of this technique, stand out the proximity between the common bile duct and the duodenal lumen, ease of identification of the dilated common bile duct, possibility of performing it in patients with ascites, and preservation of the hepatic parenchyma from the trauma resulting from the dilation.

**Figure 4:**
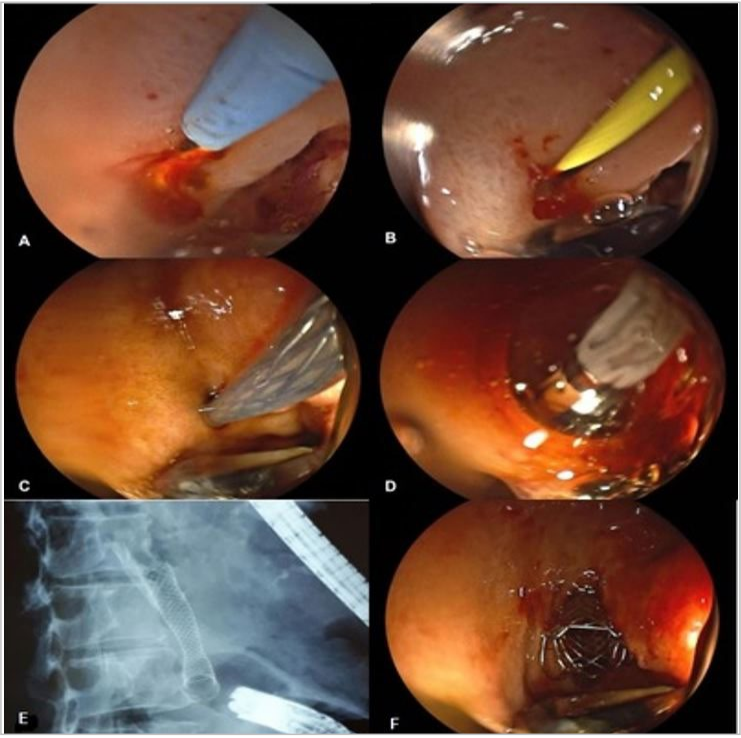
EUS-CDT. A) Endoscopic view of the 0.035” hydrophilic wire guide sustained at the puncture site; B) initial dilation of the path with a stylus; C) dilation of the path with a 6 mm hydrostatic balloon; D) passage of partially covered self-expanding metallic stent 100 mm x 60 mm; E) fluoroscopy image demonstrating aerobilia and complete drainage of bile duct contrast; F) Final appearance of the choledochoduodenostomy using the metallic stent.

Similar to the previously described techniques, dilation of the path and positioning of the prosthesis is performed with similar accessories (Figure 4).

The most frequently used stent in EUS-CDT is the fully covered self-expanding metal stent, which may or may not be associated with an inner pigtail-type plastic stent to prevent migration. However, other models of stents are also described, such as partially covered metal stents, plastic pigtail stents, and, more recently, lumen-appositing metal stents (LAMS).

The characteristics of plastic and metallic stents are deemed to be similar to those observed in biliary drainage by ERCP. Metal stents are usually thicker, giving them greater patency (about one year) when compared with plastic ones (about four months). On the other hand, plastic stents cost about 20% of the value of metal stents. Such particularities can and should be considered in a context of palliative drainage of bile duct neoplastic obstruction, which usually has a poor prognosis.

**Figure 5:**
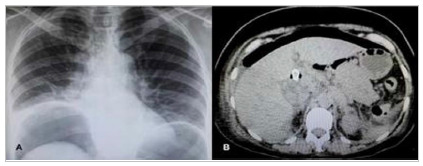
A) Chest X-ray showing pneumoperitoneum after EUS-CDT with metallic stent placement; B) Computed tomography of the abdomen showing the pneumoperitoneum and the metallic stent inside the bile duct.

Systematic reviews related to EUS-CDT demonstrate technical success of 90% to 95% and clinical success of 85% to 90%^17^. In a meta-analysis that included nine articles totaling 283 patients undergoing EUS-CDT, Hedjoudej et al18 found technical success rates of 94.6%, clinical success of 86.9%, and adverse events of 20%. Adverse events were mostly managed conservatively, the most frequent being infectious (peritonitis, cholangitis, and cholecystitis), pneumoperitoneum (Figure 5), biliary fistula, bilioma, hemorrhages, and stent migration.

Mohan et al.^19^ published a systematic review in 2019, whose primary outcome was to estimate the rate of adverse events in CDTs. The study included 572 patients, with a 13.4% risk of adverse events, the most frequent being cholangitis (4.2%), hemorrhage (4.1%), and biliary fistula (3.7%).

### Endoscopic ultrasound-guided choledochoduodenostomy versus surgical treatment

For a long time, surgical bypass of the biliary tree with hepaticojejunostomy (HJ), with or without gastrojejunostomy in cases of gastric obstruction, remained the only alternative for the treatment of neoplastic obstruction of the biliary tree in the absence or failure of ERCP. Surgery is considered an adequate option, is effective for lowering bilirubin, is definitive, and has a lower rate of reinterventions. Among its disadvantages, stand out its invasive nature, requiring longer hospital stay, morbidity of up to 35%, and mortality of up to 24%^20^. With the emergence of therapeutic alternatives, such as PTHD and EUS-BD, surgery became less and less indicated.

Artifon et al.^21^ published a prospective, randomized study in 2015 comparing EUS-CDT and HJ after ERCP failure, which included 32 patients with biliopancreatic neoplasms. The groups were statistically similar in terms of technical and clinical success rates, occurrence of adverse events, and survival rate (Table 1). There were statistical differences related to functional capacity, physical health, pain, and mental and emotional health scores (p<0.05).

### Endoscopic ultrasound-guided choledochoduodenostomy versus percutaneous transperietohepatic drainage

When ERCP fails, PTHD is a great option for clearing the bile duct. Disadvantages of this procedure include technical difficulty in patients with ascites, inconvenience of using an external drain, skin complications, and electrolyte imbalance.

Téllez-Ávila et al.^22^ published a retrospective study including 62 patients comparing PTHD and EUS-BD using different techniques. EUS-BD was superior to PTHD in terms of technical success (90% vs. 78%, p=0.03), clinical success (96% vs. 63%, p=0.04), adverse events (6.6% vs. 28%, p=0.04), length of stay (6.5 days vs. 12.5 days, p=0.009), and costs ($1,440.15 vs. $2,165.87, p=0.03).

Sharaiha et al.^23^ published a systematic review comparing PTHD and EUS-BD, totaling 482 cases. The technical success between the groups was equal. However, the echoendoscopic technique proved to be more advantageous as for the incidence of adverse events, clinical success, and need for reinterventions.

To date, only two prospective randomized studies have compared PTHD with EUS-BD. The first, published in 2012 by Artifon et al.^24^, with 25 patients (Table 2), showed statistically similar technical and clinical success, occurrence of adverse events, and costs. The second, published in 2015 by Lee et al.^25^, studied 66 patients, finding PTHD to be superior to EUS-BD in terms of technical success (96.9% versus 94.1%, p=0.008), while the echoendoscopic group had less adverse events (8.8% vs. 31.2%, p=0.022), less reinterventions (25% vs. 54.8%, p=0.015), and shorter length hospital stay (6 days vs. 12 days). The clinical success rates of the groups were 87.5% for EUS-BD and 87.1% for PTHD (p=1.0).

**Tabela 2:** Randomized studies comparing DBE versus DPTH and HJ (adapted from Teoh et al.30). EUS-BD: endoscopic ultrasound-guided biliary drainage; PTHD: Percutaneous transparietohepatic drainage; HJ: Hepaticojejunostomy.

Author	N	Technical success (%)	Clinical success (%)	Adverse events (%)	Reinterventions (%)
Artifon et al.^28^	EUS-BD: 13	100	100	15,3	-
	PTHD: 12	100	100	25	
Lee et al.^29^	EUS-BD: 34	94,1	87,5	8,8	25
	PTHD: 32	96,9	87,1	31,2 (p=0,022)	54,8 (p=0,022)
Artifon et al.^25^	EUS-BD: 14	88	71	21,42	-
	Surgical HJ: 15	94	93	13,3 (p=0,651)	

Current evidence, although limited, points to EUS-BD as having become a safe and effective alternative for clearing the biliary tract. The journal Gut, in 2018, pioneered publishing a guideline^26^ recommending the echoendoscopic approach as the first choice in cases of ERCP failure, where feasible

### Ultrasound-guided choledochoduodenostomy versus ultrasound-guided hepatogastrostomy

When opting for endoscopic treatment of obstruction of the bilioduodenopancreatic confluence, there are several feasible techniques. Current evidence considers EUS-CDT and US-HG techniques to be equally effective, although US-HG seems to have higher rates of complications, probably resulting from the dilation of gastric wall and liver parenchyma.

In 2015, Artifon et al.^27^ published a prospective, randomized clinical trial, with 24 patients undergoing EUS-CDT and 25 US-HG. They found no statistical differences regarding technical success (EUS-CDT 96% vs. US-HG 91%, p=0.6), clinical success (EUS-CDT 77% vs. US-HG 91%, p=0.23), or adverse events (16.3% in both groups).

In 2016, Khashab et al.^28^ published a retrospective, multicenter, international cohort study, comparing 60 patients undergoing EUS-CDT with 61 patients submitted to US-HG. There were no statistical differences regarding technical success (EUS-CDT 93.3% vs. US-HG 91.8%, p=0.75), clinical success (85.5% vs. 82.1%, p=0.64), and adverse events (US-HG 19.67% vs. EUS-CDT 13.3%, p=0.64). Patients undergoing US-HG had longer hospital stays (mean 5.6 days EUS-CDT vs. 12.7 days US-HG, p<0.001). The use of plastic stents was associated with a higher occurrence of adverse events (42.86% vs. 13.08%, OR 4.95, 95% CI 1.41-17.38, p=0.01). However, this data should be interpreted with caution, since there is no specification about the type of plastic prosthesis used and the incidence was calculated together for US-HG and EUS-CDT. Another factor associated with higher adverse events was the use of non-axial cautery (needle-knife) to dilate the path (OR 12.4, p=0.01), therefore the use of a cystotome being recommended.

A meta-analysis published by Khan et al.^12^ in 2015 including 1,186 patients from seven different studies showed less adverse events with EUS-CDT (OR 0.4, 95% CI 0.18-0.87). In contrast, another meta-analysis published by Uemura et al.^29^ in 2019 included 434 patients (226 undergoing EUS-CDT and 208 US-HG) and showed no statistical differences in terms of technical success, clinical success, or adverse events.

Mohan et al.^19^ published a meta-analysis in 2019 including 14 cohorts and 596 patients, aiming to compare the incidence of adverse events from EUS-CDT and US-HG. The study found no statistical differences in the occurrence of adverse events between the techniques (14.5% in EUS-CDT vs. 20.9% in US-HG, p=0.10) or between the types of stents (plastic or metal). The authors concluded that there is no clear evidence to support the recommendation for one of the techniques or for one of the stents types. 

. 

### Ultrasound-guided choledochoduodenostomy versus endoscopic retrograde cholangiopancreatography

Over time, with development and technical improvement, EUS-BD has become more effective and safer. A systematic review published in 2016 by Wang et al.^11^ showed greater technical success rates of EUS-BD in studies from 2013 onwards. With progressively more optimistic results, questions emerged as to whether EUS-BD would be safer and more effective than ERCP in the first approach to malignant obstructions of the biliopancreatic junction. Less neoplastic manipulation, lower risk of post-procedure pancreatitis, and the dispensability for long cannulation attempts are potential advantages of EUS-BD when compared with ERCP.

The three randomized studies existing so far that compared ERCP with EUS-BD were published in 2018. The first one, by Paik et al.^30^, randomized 64 patients who underwent EUS-BD (half of whom underwent EUS-CDT) and 61 who underwent ERCP. The technical/clinical results were statistically similar, but with a relevant difference (p=0.03) in the rate of complications/adverse events (6.3% vs 19.7%), such as pancreatitis (0 vs 14.8%), reintervention (5.6% vs. 42.6%), and prosthesis durability (85.1% vs. 48.9%).

In the second study, by Park et al.^31^, 15 patients underwent EUS-BD (all EUS-CDT) and 15 ERCP. Again, the technical and clinical success rates were statistically similar, but the incidence of complications with the prostheses was also similar. Interestingly, the reasons for complications were different: two cases of obstruction by food impaction and two cases of stent migration in the EUS-CDT group, and four cases of prosthesis obstruction by neoplastic growth and invasion (ingrowth) in the ERCP group.

In the third study, published by Bang et al.^32^, 33 patients were randomized to the EUS-CDT group and 34 to the ERCP one. There was no statistical difference regarding adverse events in the two groups (21.2% EUS-CDT vs. 14.7% ERCP, p=0.46).

In 2019 and 2020, three meta analyses33-35 were published endorsing that EUS-BD has a performance similar to ERCP in malignant clearance of the biliary tract, with relief of jaundice and the advantage of zero risk of post-procedure pancreatitis.

**Figura 6:**
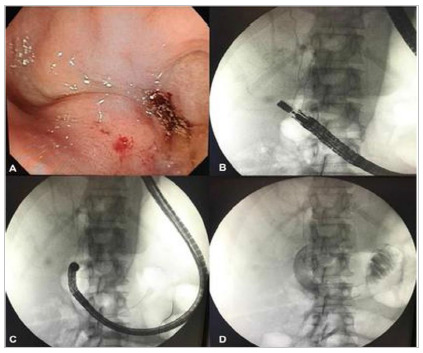
A) Endoscopic image showing neoplastic infiltration of the bulbar apex preventing the passage of the echoendoscope; B) fluoroscopic image demonstrating the adequate positioning of the metallic biliary prosthesis after DTC; C) wire pass-guide to the angle of Treitz; D) radiological control of the 9cm duodenal prosthesis with adequate passage of contrast up to the 4th duodenal portion.

Possibly, EUS-BD may soon be established as a good therapeutic option in the initial approach or indicated in cases where there are predictors of difficult ERCP (i.e., difficult biliary cannulation, tumor invasion of the papilla, and duodenal obstruction - Figure 6).

### Plastic stents versus metal stents

There is currently lack of evidence regarding the effectiveness and safety of metal versus plastic stents in EUS-CDT. The results obtained so far are often divergent and derive from meta-analyses that group different EUS-BD techniques, impairing the quality of evidence when trying to analyze the performance of each type of stent specifically in each type of therapeutic technique (i.e., EUS-CDT, US-HG, anterograde technique, and so on).

In 2018, Guo et al.^36^ published a consensus, gathering opinions from 47 experts. Regarding prostheses, the majority (87.23%) voted in favor of the metal one as the first option in EUS-BD. Two studies were used to justify the option. The first, a systematic review published by Wang et al.^11^ in 2016, which gathered 42 articles, most of which were retrospective (14 prospective), and included different EUS-BD techniques (EUS-CDT, US-HG, antegrade technique) and different types and models of stets (metal and plastic with different diameters and lengths). Although the technical and clinical successes calculated in that study were similar for both types of stents, there was a statistically significant difference in the incidence of adverse events (17.55% for metal vs. 31.03% for plastic, p=0.013).

The second, a multicenter randomized prospective study by Schmidt et al.^37^ compared a plastic stent prototype with a metal stent in patients undergoing ERCP, not including patients undergoing EUS-BD. The plastic stent had a higher incidence of dysfunction within eight weeks when compared with the metal one. It is worth mentioning though that the specific plastic biliary stent evaluated in the study is not frequently used.

The use of metal stents in EUS-BD in aim to reduce the rate of biliary fistulas was questioned during a consensus held in 2018 by specialists who were members of the Asian EUS group^26^. At the time, two studies were used as a reference to reach a decision favorable to the use of metal stents. This decision was supported by 80% of the members and considered a low level of evidence. One of the studies used was that of Gupta et al.^38^, a retrospective one with 240 patients, that found no statistic difference between the types of stents in the incidence of biliary fistula, though observing a tendency to better results with the use of the metal one. In the plastic stent group, however, there was a higher rate of complications (cholangitis), which reached statistical significance in the analysis (p=0.02). The second cited study, carried out by Khashab et al.^28^ and published in 2016, compared procedures performed in 121 patients in different centers, but with great heterogeneity in the sample number and without the correct distinction between the types of plastic stent used. As a result, a higher rate of adverse events was observed with the use of plastic stent (p=0.01) and with the use of the non-coaxial electrocautery (p=0.03).

A relevant factor in this discussion, especially in the context of Brazilian public health, is the cost difference between the two types of stents. The plastic stent costs around R$ 800.00, in contrast to the metal stent, which costs five times more, around R$ 4,000.00. This element of the discussion is often neglected, resulting in no clear recommendation when the metal stent is unavailable.

A multicenter and retrospective study by Silva^39^ in 2021 compared the use of plastic (pigtail 10fr x 07cm) and metal (100mm x 60mm) stents in 40 patients undergoing EUS-CDT and identified no statistical differences regarding technical success (95.8% metal vs 81.2% plastic, p=0.28), early clinical success (7 days: 65.2% metal vs 78.6% plastic, p=0.48), late clinical success (30 days: 90.5% metal vs 84.6% plastic, p=0.63), immediate complications (25% metal vs 12.5% plastic, p=0.21), late complications (14.3% metal vs 7.7% plastic, p=1.00), or mean survival rate (117 days vs 217 days, p=0.99)^39^.

### Lumen-apposing metal stents (LAMS)

The use of LAMS for EUS-BD was described by Binmoeller and Shah40 at the end of 2011, ten years after the introduction of EUS-BD. It is a metallic, fully covered stent, in the shape of a dumbbell and with bilateral, perpendicular phalanges for tissue anchorage.

Specifically designed for endoscopic ultrasound-guided procedures, LAMS were built on an application platform that allows the creation of the fistulous path, dilation, and introduction of the stent in a single step. The simplification of the technique can lead to an increase in the efficacy and safety of EUS-BD. In 2015, a new model of LAMS appeared, which has an improved electrocautery application system, facilitating the EUS-CDT technique. Among the advantages of LAMS in contrast to other stents are: larger lumen, which provides better drainage, reduces the risk of obstruction, and allows the passage of the endoscope through the path created for manipulation; the design of the phalanges, which allows the creation of an interface equally distributing the anchoring force between the walls of the organs, reducing the risk of bile leakage, migration, and tissue damage caused by the stent’s extremities; and complete covering that allows the removal of the stent, if necessary^41^.

A prospective multicenter study by Tsuchiya et al.^42^ followed 19 patients who underwent EUS-BD using LAMS after ERCP failure. In 100% of patients, the stent was successfully released on the first attempt, with improvement of jaundice in 95% of cases. During the observation period (mean of 145 days), stent patency was observed in 73.7% of cases, obstruction by food residues being the most common cause of reintervention.

As for EUS-BD results with LAMS, a meta-analysis published by Krishnamoorthi et al.^43^ in 2022 suggests technical success around 93% to 98% of cases and clinical success around 92% to 98%. Seven studies were included, with a total of 284 patients undergoing EUS-CDT. The occurrence of adverse events was low, reaching a maximum of 7.9%. The most frequently described adverse events are perforation, biliary fistula, bleeding, cholangitis, and abdominal pain, bleeding being the most common (2.5%).

Two other meta-analyses published in 2020 by Sanz et al.44 and Amato et al.45 compared EUS-BD using the EUS-CDT technique with the use of LAMS versus a self-expandable metal stent (SEMS). They concluded that LAMS has a high technical and clinical success rate, with no difference in complication rates, need for reintervention, or survival.

Due to the high cost, the use of LAMS is still restricted. The cost-effectiveness of LAMS versus SEMS for EUS-CDT remains to be proven. The greater technical facility makes LAMS attractive, but further studies are needed to evaluate its use as the first choice in the treatment of biliary obstruction.

## Final considerations

ERCP with placement of biliary stents remains the palliative option of choice for the treatment of malignant obstruction of the biliary tract. Drainage is possible and effective in approximately 90% of patients. In the remaining 10%, where failures occur due to multiple causes, drainage can be achieved by multiple routes (percutaneous, surgical, and endoscopic ultrasound-guided). Among the available techniques, EUS-BD, which can be performed soon after the ERCP fails, has excellent technical/clinical results and, because it is less invasive, presents reduced levels of complications. Despite being recent, EUS-BD has become the first option in several specialized services.

There are several methods for performing EUS-BD (US-HG, rendez-vous, EUS-CDT). In multiple centers, EUS-CDT is already considered the main one. In recent years, EUS-CDT has become attractive due to technical simplicity, preservation of the hepatic parenchyma, possibility of performing it in ascitic patients14,15, and lower rate of complications^15^ ^,^ ^28^.

Published studies demonstrate good technical/clinical results, regardless of the type of stent used. In theory, the characteristics and design of self-expandable metal stents (covering, increased diameter) would be responsible for reducing the rates of biliary fistula. Plastic stents, in use for a long time, have lower cost, increased availability, and similarly acceptable results. Achieving proof of the superiority of one result over the other has been an arduous and complex task.

Currently, due to greater technical ease, the use of LAMS for EUS-CDT is becoming attractive. However, its high cost and low availability keep its use restricted, and its cost-effectiveness for performing EUS-CDT still needs to be proven. New studies are needed to evaluate its use as a first choice in the treatment of biliary obstruction.

## CONCLUSION

EUS-BD, especially EUS-CDT, is being consolidated as a safe and effective alternative for malignant clearance of biliary tract in cases where drainage by ERCP is not possible or not successful. EUS-CDT is considered the technique of choice for EUS-BD, with a high success rate, both technically and clinically, regardless of the type of stent used (metallic or plastic). The use of LAMS as a first choice in EUS-CDT still needs to be evaluated.

PTHD, when performed by a highly experienced group, presents high performance and results comparable to other techniques. In specialized centers, PTHD should still be considered an excellent option for biliary drainage after ERCP failure. In the presence of advanced (non-resectable) lesions during surgery, and also in the unavailability or failure of other techniques, surgical HJ remains a viable option for biliary drainage.
